# Interprofessional education: a necessity in Alzheimer’s dementia care—a pilot study

**DOI:** 10.3389/fmed.2023.1235642

**Published:** 2023-09-22

**Authors:** Katharina Dressel, Irene Ablinger, Anna Andrea Lauer, Heike Sabine Grimm, Tobias Hartmann, Carina Hermanns, Marcus Schwarz, Tim Taddey, Marcus Otto Walter Grimm

**Affiliations:** ^1^Speech and Language Therapy, Campus Bonn/Düsseldorf, SRH University of Applied Health Sciences, Bonn/Düsseldorf, Germany; ^2^Nutrition Therapy and Counseling, Campus Rheinland, SRH University of Applied Health Sciences, Leverkusen, Germany; ^3^Experimental Neurology, Saarland University, Saarbrücken, Germany; ^4^German Institute for Dementia Prevention, Saarland University, Saarbrücken, Germany; ^5^Research Methods in Health and Social Science, Campus Gera, SRH University of Applied Health Sciences, Gera, Germany; ^6^Physiotherapy, Campus Rheinland, SRH University of Applied Health Sciences, Leverkusen, Germany

**Keywords:** interprofessional education, therapy professions, dementia care, competency-based education, learning methods, interprofessional relations

## Abstract

**Introduction:**

Interprofessional collaboration is seen as an indispensable prerequisite for high-quality health services and patient care, especially for complex diseases such as dementia. Thus, the current project aimed to extend interprofessional and competency-based education in the field of dementia care to the previously understudied therapy professions of nutrition, speech-language pathology, and physiotherapy.

**Methods:**

A three-day workshop was designed to provide specific learning objectives related to patient-centered dementia care, as well as competences for interprofessional collaboration. Teaching and learning approaches included case-based learning in simulated interprofessional case-conferences and peer-teaching. A total of 42 students (*n* = 20 nutrition therapy and counseling, *n* = 8 speech-language pathology, *n* = 14 physiotherapy), ranging from first to seventh semester, finished the whole workshop and were considered in data analysis. Changes in self-perceived attitudes toward interprofessional collaboration and education were measured by the German version of the UWE-IP. An in-house questionnaire was developed to evaluate knowledge and skills in the field of dementia, dementia management and interprofessional collaboration.

**Results:**

Participation in the workshop led to significant improvements in the total scores of the UWE-IP-D and the in-house questionnaire, as well as their respective subscales. Moderate to large effect sizes were achieved. All professions improved significantly in both questionnaires with large effect sizes. Significant differences between professions were found in the UWE-IP-D total score between students of speech-language pathology and physiotherapy in the posttest. Students of nutrition therapy and counseling revealed a significant lower level of self-perceived knowledge and skills in the in-house questionnaire pre- and post-testing.

**Discussion:**

The pilot-study confirms the effectiveness of interprofessional education to promote generic and interprofessional dementia care competencies and to develop positive attitudes toward interprofessional learning and collaboration in the therapy professions, thus increasing professional diversity in interprofessional education research. Differences between professions were confounded by heterogenous semester numbers and participation conditions. To achieve a curricular implementation, interprofessional education should be expanded to include a larger group of participants belonging to different professions, start early in the study program, and be evaluated over the long term.

## 1. Introduction

### 1.1. Interprofessional collaboration and education

Given the current challenges in health care, interprofessional collaboration, which involves regular interactions and negotiations between different health professions (1, 2), is seen as an indispensable prerequisite for high-quality health services and patient care by global leaders and in research (2–8). There is evidence that interprofessional collaboration has positive effects on clinical processes and outcomes as well as on patient reported outcomes, although clear conclusions are difficult to draw due to methodological limitations (2, 9, 10). To date, however, health care providers have typically operated under a single disciplinary approach. Interprofessional education (IPE), where “members or students of two or more professions learn with, from and about each other to improve collaboration and the quality of care and services” (11) is seen as a promising way to develop competencies associated with effective collaborative teamwork (3, 6–8). It is expected that early exposure to IPE will in turn lead to behavioral changes in future professional practice, optimizing health system performance toward the quadruple aim of enhanced patient’s health care experience, improved population health, reduced costs and improved work life of health care providers (1, 12). Comparable to professional practice, undergraduate education is predominantly organized uniprofessionally, with learners of different health professions being trained in isolation, resulting in limited knowledge and skills in interprofessional collaboration (1, 13).

### 1.2. Interprofessional collaboration and education in Germany

In Germany, too, the need for interprofessional collaboration and education has been recognized and is mentioned in some regulations governing education (6, 14–16). In particular, the IPE initiative “Operation Team” (17), funded by the non-profit Robert Bosch Stiftung, has strengthened the development and anchoring of IPE in Germany in various funding phases. There are now some interprofessional training centers and interprofessional curricula (6) [e.g., Medical Faculty of Heidelberg (18); University Medicine Berlin Charité (19); Interprofessional Healthcare of the Baden-Wuerttemberg Cooperative State University (DHBW) Heidenheim (20)], but overall training is mainly organized uniprofessionally. The situation remains difficult, especially for non-medical professions, whose primary education is predominantly at vocational schools and thus limited to lower education levels [European Qualifications Framework EQF, Level 4 (21)], with the exception of some study programs (22, 23) (see Box 1).

**BOX 1 Training of non-medical health professions in Germany**

Traditionally, initial education in non-medical health professions has been provided in three-year training programs at vocational schools with a state qualification at the end. To meet the increasing demands for high-quality health care, there is an ongoing debate, whether and to what extent the education of non-medical health professions should be academized, raising non-medical education programs from upper secondary level EQR level 4 (21) to the bachelor’s degree level EQR level 6 (21, 23). In 2020, the discussion ended in the full academization of midwifery training (24), and a partial academization for nursing studies (16), with most trainees completing vocational schools and some graduating from university for better career opportunities and professional practice on scientific basis. For the therapy professions, a model was introduced in 2009 that allows primary education at university level (25). As a result, a variety of training programs have evolved, ranging from pure vocational schools, to studies combined with vocational schools, to pure higher education at university. The final decision about education in the therapy professions is expected in 2024, with the revision of profession-specific legislation and regulations, of which interprofessionality is an essential component (5, 23, 26).

### 1.3. Competencies for interprofessional collaboration

In contrast to profession specific and generic competencies for all health professions, which can also be acquired uniprofessionally, interprofessional collaborative competencies can only be achieved through IPE (4). In international frameworks these competencies include role clarification, team functioning, and interprofessional communication, but also values and ethics, conflict resolution, reflection and patient-centered care (4). These collaborative competencies are acquired in three stages: in the first phase (exposure) students gain a deeper understanding of their own discipline and a first insight into the roles of other health care providers, challenging misconceptions about professional roles; in the second phase (immersion), interprofessional role learning takes place through collaborative interactions; in the final phase (mastery) dual professional identity is mastered (13, 27, 28). To develop interprofessional competencies, IPE should start early and be continued throughout the course of studies (7, 8, 13), although the optimal timing is still under debate (29). If IPE is introduced before one’s professional identity has developed, students may not be ready for collaborative learning. A late start may reinforce stereotypes toward other professions (29).

The competencies acquired in the three stages of IPE can be classified on six levels according to Barr’s et al. modified Kirkpatrick model (30). Level 1 captures the learner’s reaction to the IPE experience, level 2a comprises the modification of attitudes between participating professions and toward the value of interprofessional collaboration for patient-centered care, and level 2b covers the acquisition of knowledge and skills associated to IPE. Levels 3, 4a and 4b are related to the individual transfer of IPE into practice, changes in organizational practice and to improvements in clinical outcomes.

### 1.4. Evidence for interprofessional education

Although the evidence base of IPE is still challenging (6), reviews demonstrate positive effects of IPE, especially among undergraduate learners in the IPE learning stages 1 und 2 (13) and in outcomes associated with level 1, 2a, 2b (31). IPE leads to changes in students’ perception and attitudes toward collaborative learning and practice (8, 31–34). Most reviews also found improved knowledge and skills (e.g., understanding the roles of other disciplines, communicating with other professions) following IPE intervention (31, 32), with Spaudling et al. (8) reporting ambiguous results. There is a growing body of evidence on the successful translation of IPE to collaborative professional practice and patient outcomes, but studies are less common, and results should be interpreted with caution (7, 8, 31).

IPE studies generally comprise six to 10 professions, but also a broader and smaller professional mix (13, 31). Lairamore et al. (35) compared a case-based IPE event with five and 10 different professions, with the smaller group having an advantage due to the more focused case construction. Nursing, medicine (13, 31) and, within the therapy professions, physiotherapy (13) are the most frequently included professions in IPE studies. Other professions, as nutrition therapy and counseling and speech-language pathology are still underrepresented, which leads to the demand of more diversity in IPE and the establishment of IPE beyond medical faculties (6).

Most IPE studies assess the success of their intervention with self-report surveys (8, 29). While there exist numerous instruments internationally, there are only a few translated into German (36), among them the University of the West of England Interprofessional Questionnaire UWE-IP (37–39). It measures self-perceived attitudes toward interprofessional learning, interaction and relationships and communication and teamwork. The UWE-IP shows good psychometric properties. The underlying factor structure is considered good. However, a relatively high correlation between some scales is shown, challenging the assumption of different dimensions (37). The UWE is recommended for the evaluation of IPE programs and allows the comparison across studies (6, 36). Nevertheless, the exploration of an IPE intervention normally requires more than one assessment tool and the combination of different evaluation methods (6).

### 1.5. Interprofessional dementia education

Due to the multi-layered components of dementia, person-centered, interprofessional approaches can increase the preventive or therapeutic potential in people with dementia (40–43). This is coupled with the need for collaborative coordination in dementia care to ensure optimal support for those affected (44–48). However, interprofessional collaborative practice in dementia care is still rare (49, 50). In this context, IPE can pave the way to prepare the future dementia work force for the delivery of integrated care (44, 46).

The format of IPE in dementia care varies from (extra-)curricular under- and postgraduate programs of different length (45, 48, 51–54), including online and technology-based education formats that allow for synchronous and asynchronous elaboration of teaching contents (45, 52, 53). In general, interprofessional dementia education resulted in increased knowledge about dementia and improved attitudes and empathy toward persons with dementia and their carers (44, 48, 51, 53–55). Regarding interprofessional collaborative competencies, the majority of the studies focused on the modification of attitudes, knowledge and skills (Barr et al. level 2a, 2b) (44, 45, 53–57), mostly with positive findings. Some studies even achieved medium to large effect sizes (45, 48), but the overall quality of the methodology is considered low (44).

### 1.6. Objectives

So far, the therapy professions are still underrepresented in IPE research. Their inclusion is urgently needed, in Germany especially in the context of the efforts to academize non-medical education programs, and globally in the context of complex diseases as dementia. Therefore, the current pilot study aimed to extend IPE to the hitherto less considered study courses of nutrition therapy and counseling, speech-language pathology and physiotherapy in the field of dementia care at a German university of applied health sciences, that offers education beyond the primary professional qualification. More specifically, the pilot study was conducted to improve generic competencies related to dementia and person-centered dementia management as well as to changes in attitudes, knowledge and skills related to interprofessional collaboration (Barr et al. level 2a, 2b) in general and in dementia care. We hypothesized that the participation would lead to positive changes in self-perceived attitudes toward interprofessional collaboration and education, measured with the UWE-IP-D (37), and would result in improvements associated with knowledge and skills in the field of dementia, dementia management and interprofessional collaboration, measured with an in-house questionnaire. We did not expect any differences in gains between the individual health professions.

## 2. Methods

### 2.1. Participants

Our intervention was designed as an interprofessional workshop for students of the bachelor’s degree programs in physiotherapy, nutrition therapy and counseling and speech-language pathology at the SRH University of Applied Health Sciences. The students studied at different SRH locations in Nordrhein-Westfalen (North Rhine-Westphalia, NRW) and met in November, shortly after the start of the winter semester, for a joint workshop at the SRH Campus Rheinland. We started the workshop with 53 students (*n* = 28 nutrition therapy and counseling, *n* = 8 speech-language pathology and *n* = 17 physiotherapy). A total of 42 students finished the whole workshop and were considered in data analysis (Table 1, participants). Reasons for exclusions were participation in not all three workshop days, missing information on the questionnaires, so that an allocation pre/post was not possible and missing submission of the questionnaire. All participating students received credits points for their study program, no other incentives were given. The workshop was facilitated by some of the authors, who taught in the individual study programs: three experienced nutrition therapy and counseling professors and senior assistants, two speech-language pathology professors (one of whom participated online for health reasons), and one assistant for physiotherapy. Among the teaching stuff, one had previous experience in interprofessional education. All participants gave their consent to the further use of the collected data in anonymized form. The study was reviewed and approved by the SRH University of Applied Health Sciences for ethical standards.

**Table 1:** Participants.

Study program	*n*	Semester (*n*)	Prior knowledge	Mode of delivery
Nutrition therapy and counseling	20	1 (14)	No dementia specific knowledge; IPE: no prior experience	Obligatory
		3 (4)	Basic subject specific knowledge in dementia; IPE: no prior experience	
		5 (2)	Basic subject specific knowledge in dementia; IPE no prior experience	
Speech and language pathology	8	7	Basic subject specific knowledge in dementia	Voluntary as part of an elective module, in which students could choose between different thematic offers
			IPE: No prior experience	
Physiotherapy	14	7	Basic subject specific knowledge in dementia	Obligatory
			IPE: No prior experience	

### 2.2. Interprofessional education workshop

We used a pre-post design to evaluate the IPE intervention. Data were collected immediately before and after a three-day workshop. Our design did not take a control group or randomization into account, as the workshop was part of ongoing courses in each degree program. The workshop consisted of a total of 30 lessons of 45 min each, with a one-day break between the second and third workshop day.

The workshop was conceived as a pilot project with the aim of implementing IPE in the curriculum of bachelor health degree programs in the future. Due to the high relevance for all professions, the topic of person-centered dementia care in an interprofessional setting was chosen for the workshop at the SRH Campus Rheinland. Interprofessional learning was aligned to the learning stages one (exposure) and two (immersion) with first insights into the roles of other health care providers and interprofessional role learning (13, 27). Learning objectives related to generic competencies in knowledge and skills about dementia and dementia management as well as to the framework of Barr’s et al. modified Kirkpatrick model, primarily Level 2a, 2b (changes in attitudes, knowledge and skills related to interprofessional collaboration) (30), providing competences for interprofessional collaboration in relation to one’s own and other professionals’ roles and responsibilities, teamwork and communication. Some of these collaborative competences were formulated specific for dementia, and some described interprofessional collaboration and education in general. Examples of learning objectives can be found in Table 2.

**Table 2:** Examples of main learning objectives.

Area	Examples of learning objectives
Knowledge and skills in dementia and dementia management	Explaining the molecular mechanisms of Alzheimer’s Disease
	Planning intervention according to the ICF for persons with dementia
	Describing language and communication disorders and disorders of food intake in people with dementia
	Explaining the importance of physical activity for people with dementia
Interprofessional learning and collaboration in general	Recognizing the value of interprofessional learning in relation to interprofessional team functioning, communication, and role clarification
	Valuing the expertise of other health professions
	Describing the scope of practice of other health professions
	Passing specialist information in an understandable way in the interprofessional team
Interprofessional learning and collaboration in dementia care	Recognizing the value of interprofessional collaboration in dementia management
	Identifying interfaces of different health professions in dementia management
	Setting treatment goals with other health professions relevant for a specific person with dementia
	Clarifying responsibilities in dementia management

Derived from the focused competences, teaching and learning approaches included case-based learning (13, 58) in simulated interprofessional case-conferences (13, 59–61) and peer-assisted learning for knowledge transfer among different health professions and across different semester levels (62–64). These are commonly used pedagogical approaches in IPE and health education, relying on teamwork and allowing the combination of collaboration, simulation of real-life scenarios, concrete experiences and reflection (13, 65). According to the IPE idea students should learn with, about and from each other. Small group and active learning methods (e.g., poster walks) are other key components of IPE to foster active involvement and socialization in a safe and non-hierarchical atmosphere (66). Short input sequences from teaching stuff were only used to introduce new topics (e.g., information about interprofessional collaboration and education; the use of scaffolds for case-conferences). The main task of the lecturers was to observe the learners in a structured way and to identify and support teachable moments and to moderate plenum discussions. Additionally, sufficient breaks and social events were provided for the personal and professional exchange of the participants.

The program focused on three main topics, each of which was addressed on one of the three workshop days. (1) Understanding of roles. (2) Collaborative dementia management. (3) Simulated interprofessional case-conference with role-play and development of a multicomponent treatment approach. All other materials (e.g., PowerPoint slides of the input sequences, scaffolds, results of group work) were accessible to all students during or after the workshop by email. The workshop was held as planned. Table 3 summarizes the content of the interprofessional dementia workshop.

**Table 3:** Content of the interprofessional dementia workshop.

	Main topic	Group composition, content and used methods
Day 1	Understanding of roles Generic competencies IP Competencies: Barr 2a, b Learning stage 1, 2	Evaluation Warming up: speed dating for first contact KL: interprofessional education; quality criteria in literature research IP-groups: responsibilities and boundaries of individual professions in general PL-discussion: understanding of professional roles and ethics Peer teaching in IP-groups to specific themes: ICF in general and in dementia (tutors physiotherapy, speech-language pathology; tutees nutrition therapy and counseling); molecular mechanisms in dementia (tutors nutrition therapy and counseling; tutees physiotherapy, speech-language pathology); posters were prepared uniprofessionally before the workshop PL-discussion: debriefing
Day 2	Collaborative dementia management Generic competencies IP competencies: Barr 2a, b IP learning stage 1, 2	UP-groups: professions in dementia management, responsibilities and therapy; review of the literature and poster preparation IP-groups: poster walk—discussion of dementia-management results; overlaps in therapy professions Preparation of Day 3: KL, scaffolds for case conferences (ISBAR, ICF-oriented guideline for case-conference) and for observation protocols; introduction to case study PL-discussion: debriefing Social event to connect
Day 3	Simulated interprofessional case-conference with role-play IP competencies: Barr 2a, b IP learning stage: 2	KL: feedback rules and feedback methods UP-groups: uniprofessional preparation of the case study IP-groups: ICF-oriented case-conference with role play; feedback and reflection (process, professional exchange, communication etc.) a case presentation: general clinical history for all study programs; additional discipline-specific information, only accessible to the respective health profession (e.g., information about language and communication abilities only for speech-language pathology students) PL-discussion: debriefing IP-groups: drafts of a multicomponent therapy for dementia PL-discussion: presentation of results PL-discussion: debriefing, closure Evaluation

KL, Key lectures in the plenum; IP, Interprofessional; IP-groups, Interprofessional group compositions à 8 students; PL-discussion, Plenum discussion; UP-groups, Uniprofessional group compositions à 8 students.

### 2.3. Outcomes and measures

We used the German version of the UWE-IP (UWE-IP-D) (37–39) to measure self-perceived interprofessional attitudes immediately before and after our workshop in the domains of communication and teamwork (*Communication and Teamwork Scale*, 9 items), interprofessional learning (*IP Learning Scale*, 9 items), interprofessional interaction (*IP Interaction Scale*, 9 items) and interprofessional relationships (*IP Relationship Scale*, 8 items). The UWE-IP-D is a reliable psychometrically validated instrument (37). The 35 Items were rated on a 4-point (*Communication and Teamwork Scale*) or 5-point Likert scale with scores representing “strongly agree” to “strongly disagree.” Depending on the number of points achieved attitudes can be classified as positive, neutral or negative attitudes (37). In all subscales a lower score relates to a more positive response.

An in-house questionnaire (*n* = 33 items) was developed to assess self-reported acquisition of knowledge and skills related to interprofessional teamwork in dementia management (*Domain IP Teamwork in Dementia Management*, 10 items), generic knowledge and skills on dementia and patient centered dementia care (*Domain Knowledge and Skills on Dementia Care* 8 items) and to interprofessional communication skills (*IP Communication*, 12 items) pre- and post-training. Items were measured on an 8-point Likert scale with 1–2 = “is completely true,” 3–4 = “is true,” 5–6 = “is partly true,” 7–8 = “is not true.” Three questions were addressed exclusively in the post-evaluation. Here, the quality of the workshop and the influence of the workshop on the future cooperation between the professional groups had to be scored on a 5-point scale, with one being the best rating. The last item offered the possibility of an open evaluation of the workshop. The students were able to comment on what they particularly liked about the workshop and what they had to criticize.

### 2.4. Analyses

For data analysis, the IBM SPSS Statistics 28 software was used. Missing data were compensated by mean value substitutions (<1%). According to Mahler et al. (37) we recoded some UWE-IP-D questions in reversed order. For further analysis, we relied on the sum scores, with a minimum of 9 points and a maximum of 36 points for the *Communication and Teamwork Scale*, a minimum of 9 and a maximum of 45 points for the *IP Learning Scale* and *IP Interaction Scale* and a minimum of 8 points and a maximum of 40 points for the *IP Relationship Scale*. According to the points achieved, attitudes were classified as positive, neutral or negative. This corresponded to scores of 9 to 20, 21 to 25 and 26 to 36 in the *Communication and Teamwork Scale*, scores of 9 to 22, 23 to 31 and 32 to 45 in the *IP Learning Scale* and *IP Interaction Scale* and scores of 8 to 20, 21 to 28 and 29 to 40 in the *IP Relationship Scale* (37–39). Analyses on the in-house questionnaire were also evaluated based on the sum scores. 30 items were included in the quantitative analyses, resulting in a total possible score of 30 to 240 points, a score of 10 to 80 for the *Domain IP Teamwork in Dementia Management*, a score of 8 to 64 for the *Domain Knowledge and Skills on Dementia Care*, and a score of 12 to 96 for the *Domain IP Communication*. Lower values indicate high self-perceived knowledge and skills, and higher values poorer self-assessment.

Pre-post analyses on mean sum scores of the UWE-IP-D and the in-house questionnaire were calculated for the whole group using the paired-samples t test. Repeated measures analyses of variance (ANOVA) were performed with time as the within-factor and profession as the between-factor. *Post hoc* pairwise comparisons between professions were examined with paired-samples t tests. Tukey’s correction was applied to control for potential alpha inflation due to repeated measurements. Except for the profession comparisons, all statistical tests were calculated on the bootstrap procedure, with each 1,000 simulated sample draws, to compensate for deviations from the requirements for a normal distribution of the analyses. Two-tailed *p*-values and alpha levels of 0.05 were used for all statistical tests. Furthermore, effect size measures were computed according to *Hedges’g*, with g > 0.8 considered as a large effect, g > 0.5 as a moderate effect, and g > 0.2 as a small effect (67, 68). Cronbach’s alpha was used to assess the internal item consistency of the three dimensions in the in-house questionnaire. Three items from the in-house questionnaire were used for the qualitative analysis. The evaluation of these open-ended responses followed the principles of qualitative analysis (69). Responses were transcribed by one of the authors and subsequently content was grouped and coded by two raters.

## 3. Results

### 3.1. UWE-IP-D

**Figure 1:**
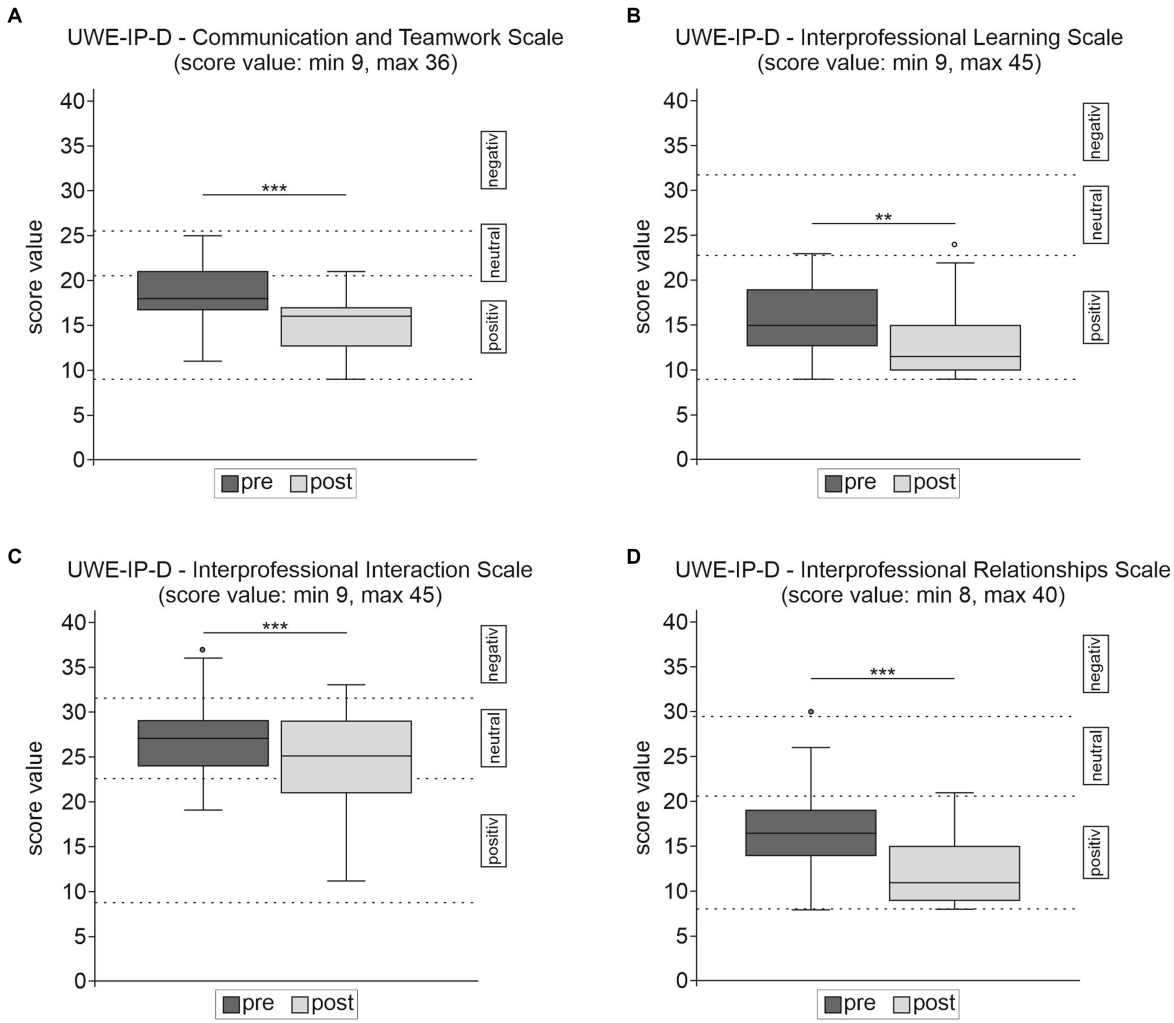
Results of the UWE-IP-D sub-scales **(A–D)** for the whole sample (*n* = 42) pre- and post-intervention. Cumulative Scores for the UWE-IP-D were attributed to positive, neutral, negative areas according to Pollard et al. (38, 39). ^***^p ≤ 0.001 and ^**^p ≤ 0.01. In all subscales a lower score relates to a more positive response.

A total of 42 students completed the pre-post comparison with the UWE-IP-D (37). An analysis across all students showed that participation in the interprofessional dementia care workshop led to significant overall improvements in the total UWE-IP-D score (Mean score difference 13.86, 95% CI 10.93, 16.95, *p* < 0.001) and in all four subscales (*Communication and Teamwork Scale:* Mean score difference 3.21, 95% CI 2.33, 4.19, *p* < 0.001; *IP Learning Scale*: Mean score difference 2.71, 95% CI 1.48, 4.12, *p* = 0.002; *IP Interaction Scale*: Mean score difference 3.05, 95% CI 1.67, 4.38, *p* < 0.001 and *IP Relationship Scale*: Mean score difference 4.88, 95% CI 3.62, 6.14, *p* < 0.001) with moderate to large effect sizes from *Hedges’g* 0.59 to 1.19. Table 4 summarizes the results in the UWE-IP-D questionnaire. In Figure 1 the median with classified attitudes of the whole group is shown for all subscales.

**Table 4:** Results of the UWE-IP-D subscales pre- and post-intervention for the whole sample (*n* = 42).

		Mean sum score	SE	Differences	95% confidence interval		Value of *p*	Hedges’ g
				Mean sum scores	Lower value	Upper value		
Total	T1	77.75	1.48	13.86	74.72	80.89	<0.001	1.34
	T2	63.89	1.65		60.76	67.18		
*Communication and Teamwork Scale*	T1	18.20	0.49	3.21	17.21	19.15	<0.001	0.91
	T2	14.99	0.49		14.01	15.97		
*Interprofessional Learning Scale*	T1	15.57	0.62	2.71	14.38	16.76	0.002	0.59
	T2	12.86	0.59		11.71	14.05		
*Interprofessional Interaction Scale*	T1	27.19	0.64	3.05	25.90	28.48	<0.001	0.64
	T2	24.14	0.84		22.43	25.76		
*Interprofessional Relationships Scale*	T1	16.79	0.64	4.88	15.57	18.05	<0.001	1.19
	T2	11.90	0.53		10.90	13.02		

SE, Standard error of the mean value; Maximal total score value = 166. Maximal score value in *Communication and Teamwork Scale* = 36, in *Interprofessional Learning Scale* = 45, in *Interprofessional Interaction Scale* = 45, in *Interprofessional Relationships Scale* = 40.

ANOVA revealed a significant main effect in the overall mean sum score values for time [*F*(1, 39) = 84.43, *p* < 0.001, partial η^2^ = 0.68], but not for profession [*F*(2, 39) = 1.78, *p* = 0.181]. There was a statistically significant interaction between time and profession [*F*(2, 39) = 4.92, *p* = 0.012, partial η^2^ = 0.202]. The overall mean sum scores did not differ significantly according to professional groups before the intervention [*F*(2, 39) = 0.29, *p* = 0.750, η^2^ = 0.015], but after the intervention [*F*(2, 39) = 4,792, *p* = 0.014; η^2^ = 0.197]. *Post hoc* analysis revealed a significant difference in the mean UWE-IP-D total score after intervention only between students of speech-language pathology and physiotherapy (Mean difference 13.09, SE 4.38, *p* = 0.013). Regardless of this, all professions improved significantly in the UWE-IP-D total score after intervention [nutrition therapy and counseling (Mean score difference 16.23, 95% CI 12.12, 20.24, *p* < 0.001), speech-language pathology (Mean score difference 18.88, 95% CI 11.13, 27.50, *p* = 0.005) physiotherapy (Mean score difference 7.59, 95% CI 4.31, 10.67, *p* = 0.002)], each with large effect sizes ranging from *Hedges’g* 1.18 to 1.65.

### 3.2. In-house questionnaire

Self-reported acquisition of interprofessional teamwork in dementia management, and generic dementia specific knowledge and skills and interprofessional communication were assessed with our in-house questionnaire. Data from 41 students were included in the quantitative in-house questionnaire evaluation. All three domains of the in-house questionnaire revealed reasonable internal consistency with Cronbach’s alpha at both testing points, ranging from 0.91–0.95 (*Domain IP Teamwork in Dementia Management*) to 0.72–0.90 (*Domain Knowledge and Skills on Dementia Care*), and 0.91–0.96 (*Domain IP Communication*), respectively.

Overall significant results were achieved in the total in-house questionnaire score (Mean score difference 62.89, 95% CI 50.92, 74.86, *p* < 0.001) and in all three domains [*Domain IP Teamwork in Dementia Management* (Mean score difference 25.49, 95% CI 20.30, 30.68, *p* < 0.001), *Domain Knowledge and Skills on Dementia Care* (Mean score difference 21.84, 95% CI 18.25, 25.44, *p* < 0.001), *Domain IP Communication* (Mean score difference 15.56, 95% CI 11.22, 19.90, *p* < 0.001)], with large effect sizes from *Hedges’g* 1.12 to 1.90. Table 5 provides an overview of the results of the in-house questionnaire.

**Table 5:** Results of the in-house questionnaire domains pre- and post-intervention for the whole sample (*n* = 41).

		Mean sum score	SE	Differences	95% confidence interval		Value of *p*	Hedges’ g
				Mean sum scores	Lower value	Upper value		
Total	T1	131.48	5.25	62.89	121.64	142.35	<0.001	1.64
	T2	68.59	4.49		60.01	77.42		
*IP Teamwork in Dementia Management Scale*	T1	48.02	2.12	25.49	44.06	52.30	<0.001	1.54
	T2	22.54	1.57		19.63	25.71		
*Knowledge and Skills on Dementia Care Scale*	T1	42.05	1.62	21.84	38.78	45.46	<0.001	1.90
	T2	20.20	1.39		17.68	23.15		
*IP Communication Scale*	T1	41.41	2.16	15.56	37.49	45.91	<0.001	1.12
	T2	25.85	1.82		22.34	29.41		

SE, Standard error of the mean value; Maximal total score value = 240. Maximal score value in *Domain IP Teamwork in Dementia Management* = 80, in *Domain Knowledge and Skills on Dementia Care* = 64, in *Domain IP Communication* = 96.

**Figure 2:**
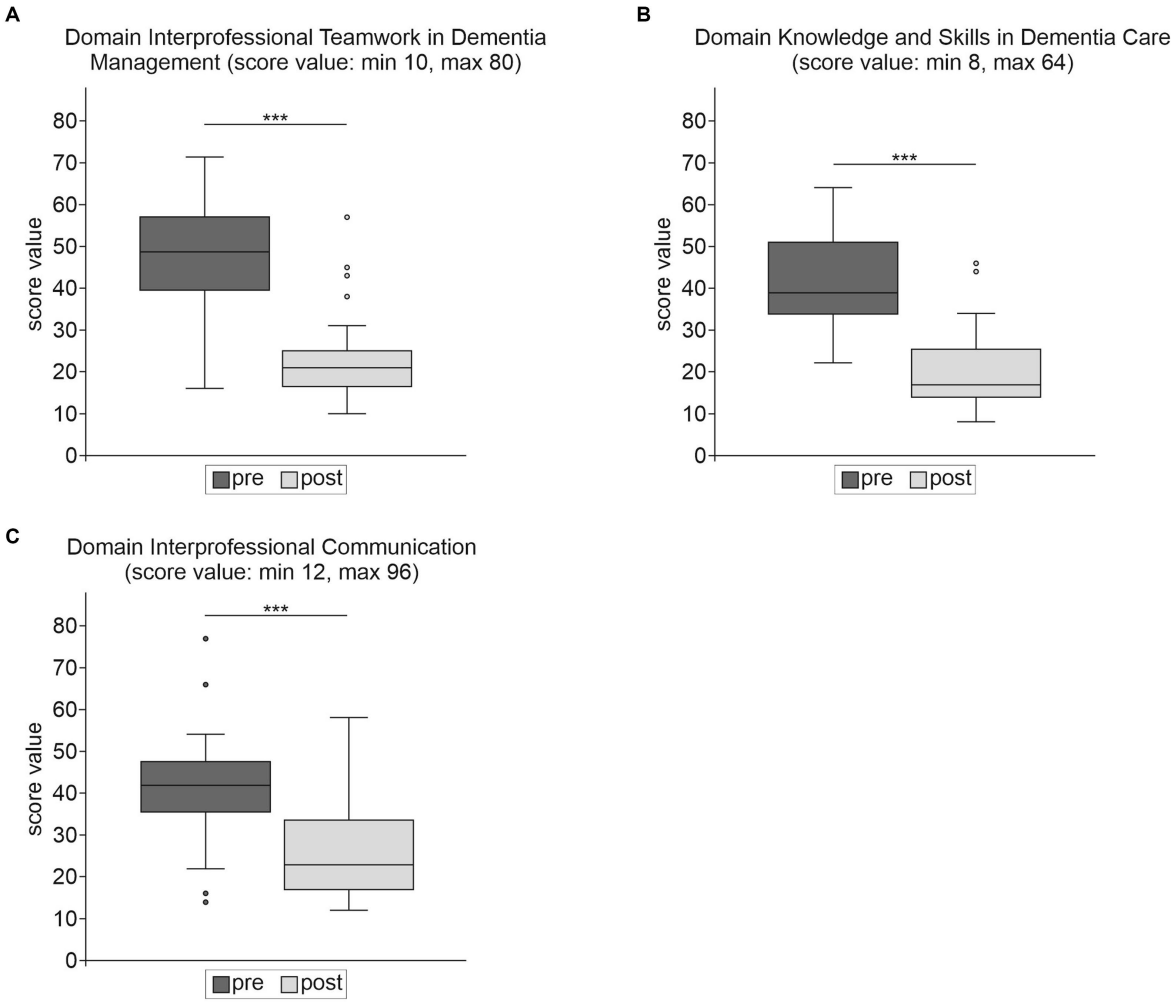
Results of the in-house questionnaire domains **(A–C)** for the whole sample (*n* = 41) pre- and post-intervention. ^***^p ≤ 0.001 and ^**^p ≤ 0.01. In all domains a lower score relates to a more positive response.

The median in the *Domains IP Teamwork in Dementia Management* and *Knowledge and Skills on Dementia Care* changed from moderate ratings before the intervention to positive ratings after the intervention. Self-perceived attitudes in the *Domain IP Communication* were at the border between moderate-positive ratings in the pre-test and positive in the post-test (see Figure 2).

ANOVA revealed significant main effects in the overall mean sum score values for time [*F*(1, 38) = 103.473 *p* < 0.001, partial η^2^ = 0.731] and for profession [*F*(2, 38) = 9.702, *p* < 0.001, partial η^2^ = 0.338]. There was no statistically significant interaction between time and profession [*F*(2, 38) = 1.154, *p* = 0.326, partial η^2^ = 0.057]. Overall results on the in-house questionnaire differed significantly according to professional groups before the intervention [*F*(2, 38) = 5.907, *p* = 0.006, η^2^ = 0.237] and after the intervention [*F*(2, 38) = 5.812, *p* = 0.006, η^2^ = 0.234]. *Post-hoc* analyses revealed that students of nutrition therapy and counseling had marginally but non-significant higher mean sum scores than students of speech-language pathology (Mean score difference 30.20, *p* = 0.057) and significantly higher scores than students of physiotherapy (Mean score difference 33.46, *p* = 0.008) in pre-testing, and compared to students of speech-language pathology (Mean score difference 36.77, *p* = 0.005) in post-testing. This indicates a lower level of self-perceived knowledge and skills.

All professions improved significantly in the total score of the in-house questionnaire [nutrition therapy and counseling (Mean score difference 67.16, 95% CI 44.38, 86.25, *p* < 0.001), speech-language pathology (Mean score difference 73.72, 95% CI 61.85, 84.92, *p* < 0.001) physiotherapy (Mean score difference 50.91, 95% CI 36.65, 65.50, *p* < 0.001)], each with large effect sizes ranging from *Hedges’g* 1.37 to 3.72.

### 3.3. Workshop feedback

Of the students who participated, 98% felt that the IPE workshop helped to improve collaboration between disciplines, 84% thereof unrestricted. On a 5-point evaluation scale of the workshop, with 1 being the best rating, the average rating for the entire sample was 1.7. Students of speech-language pathology evaluated the workshop with a mean of 1.2, students of nutrition therapy and counseling rated it 1.4 and students of physiotherapy rated it 2.4. In open questions, students indicated that they particularly appreciated the open exchange and teamwork between the professions. Some of them separately mentioned the case study positively. The students benefited above all from the collegial exchange between the professional groups, the dementia-specific increase in knowledge, but also from the fact that they received general information about and from other professional groups and about professional interfaces. In addition, there was a desire to expand the workshop to include other diseases and professions. However, some few students also wanted more student input, homogeneous groups in terms of study duration and more involvement of physiotherapy students.

## 4. Discussion

The therapy professions have received little attention to date in IPE research in general and in dementia care programs (6, 44, 48, 51, 53). Therefore, an IPE pilot workshop was designed for the three therapy professions of nutrition therapy and counseling, speech-language pathology, and physiotherapy. The three-day workshop was integrated into ongoing university courses. Like most studies (8, 13, 31, 44), our IPE program was situated in the IPE learning phases 1 and 2 [first insights into the roles of other health professions, interprofessional role learning through collaborative interactions (27, 28)] with outcomes related to Barr et al. levels 2a, 2b (30).

### 4.1. Summary and interpretation of the UWE-IP-D and in-house questionnaire results

We observed significant positive changes in attitudes toward other professions and toward the value of IPE, which is in accordance with the IPE literature in general (8, 31, 34) and in the field of dementia education (44, 45, 53, 54, 57). Attitudes measured with the UWE-IP-D Scales *Communication and Teamwork, IP Learning*, and *IP Relationship* (37) were already positive before training and improved significantly with moderate to large effect sizes after the workshop, indicating high willingness and motivation to collaborate and learn together. Gains on these three UWE-IP Scales were also demonstrated in other studies (70–74). The most negative ratings pre- and post-training were on the *IP Interaction Scale*, related to status, stereotypes and inequality among professions, although there was a significant reduction of negative perceptions after the workshop. The worse rating in the *IP Interaction Scale* is consistent with other studies using the UWE-IP (39, 71, 72, 74–76), but only some of these observed improvements after an IPE program as we did (71, 72). The students’ views regarding interprofessional interaction may be influenced by notions of imbalances in the hierarchy of the health care system (31), which in turn supports the claim of introducing IPE early in health education before negative stereotyping is reinforced (7, 8, 13, 29).

There is a general discussion in the literature about the need to test the effectiveness of the IPE intervention at different levels (6, 77). While standardized testing procedures, such as the UWE-IP (37, 39), allow for international comparisons of individual projects, the used evaluation instruments should also be adapted to the respective IPE settings and contents, so that as many aspects of the intervention as possible can be covered (6, 31). Therefore, we designed an in-house questionnaire to assess self-perceived abilities of interprofessional teamwork in dementia management, generic dementia specific knowledge and skills, and interprofessional communication in more detail. Significant gains with large effect sizes were seen in competencies that can only be acquired through IPE (4) (e.g., *Domains IP Teamwork in Dementia Management*; *IP Communication*), as well as in generic competencies related to dementia specific knowledge and skills, which are usually taught uniprofessionally (*Domain Knowledge and Skills on Dementia Care*). As far as the *IP Communication* is concerned, overall rating was already moderate to positive before the intervention. Regarding self-reported generic knowledge and skills related to dementia (*Domain Knowledge and Skills on Dementia Care*) and interprofessional teamwork in dementia management (*Domain IP Teamwork in Dementia Management*), significant changes in each domain were observed, with medium ratings pre-intervention and positive ratings post-intervention. The gains are in line with other studies investigating dementia knowledge and skills before and after training (48, 53, 78, 79), except for the study of McCaffrey et al. (55), who could only find a numerical, non-significant knowledge increase. However, it should be noted that self-report instruments can reflect acquired knowledge and skills only to a limited extent (6). We are aware of only some studies in dementia care that have objectively examined the effects of IPE on knowledge gains by already published tools (48, 79) or specifically developed ones (55). Therefore, it seems promising to develop a program that teaches interprofessional collaboration in general and in dementia care while enabling the acquisition of generic dementia knowledge and including objective and self-assessment testing procedures.

### 4.2. Influencing factors on IPE

Several presage factors may have contributed to the significant effects on attitudes toward interprofessional collaboration and education as well as on dementia related knowledge and skills. Among the student characteristics, the high willingness and motivation for collaborative learning, that we had observed prior to the training, was probably conducive (31). The high proportion of female students may also have influenced the results. While Reeves et al. (31) reported mixed effects of gender in their review, Wang et al. (34) observed more positive responses in female participants compared to males. Due to the small number of male students, we did not link gender to the data of our questionnaire to ensure anonymity. Therefore, a gender-specific analysis was not possible. In addition, a climate of safety, as we provided in small learning islands, facilitator input and debriefing, and informal networking opportunities may have fostered positive IPE experiences (31). The inclusion of only three professions allowed us to construct a focused case story. Lairamore et al. (35) observed a stronger impact of a case-based IPE event in groups of five professions compared to 10 with broadened case scenarios and less involvement of the individual professions. Nevertheless, the inclusion of more professions is desirable in the future to increase the complexity of the learning situations and to stimulate transfer to real practice (80). In the qualitative feedback students wished IPE to be strengthened by the inclusion of other diseases and additional professions. Knowledge of other health providers in general and their role in dementia management, exchange within the jointed groups, and collegial interaction were seen as key personal outcomes of the workshop.

Approaches to learning and teaching are important process factors that affect IPE (6, 31), and learning activities, desired outcomes and their assessment should be adequately aligned (4, 13). In accordance with international methods, the incorporation of peer-assisted learning with tutors and tutees from different professions (64) was designed to compare and contrast professional roles and responsibilities, to gain knowledge about dementia and dementia management. Competencies related to teamwork, communication and patient-centered dementia care were additionally maximized through experiential learning (13, 65, 80, 81), with the elements of a uniprofessionally prepared case study to be negotiated in a simulated interprofessional case-conference, followed by discussion and reflection.

### 4.3. Profession specific results

Contrary to our expectations, we observed some profession specific differences. In the pretest, attitudes measured with the UWE-IP-D (37) were comparable in all three groups, but nutrition therapy and counseling had a significantly lower baseline mean score in the in-house questionnaire. These differences can possibly be attributed to the lower semester numbers of nutrition therapy and counseling students (nutrition therapy and counseling: first to fifth semester; physiotherapy and speech-language pathology: seventh semester). Regarding the UWE-IP-D, a significant interaction between time and profession was observed, as physiotherapy students had lower pre-post gains compared to the other two professions. The skeptical attitude of some physiotherapy students toward the event is also reflected in the student feedback: physiotherapy students evaluated the workshop with 2.4, while the mean rating of speech-language pathology students and nutrition therapy and counseling students was 1.2 and 1.4, respectively. All three professions found that the workshop improved interprofessional collaboration in dementia management, but some participants wished physiotherapy students to be more included. The differential number of semesters can only partially explain the observed imbalances, since students of physiotherapy and speech-language pathology were both in the seventh semester. Another reason may lie in the fact that the workshop was led by several experienced nutrition therapy and counseling and speech-language pathology professors and senior assistants, whereas only one assistant was available for physiotherapy for organizational reasons, leading to unbalanced professional representatives in the mixed small groups. According to Reeves et al. (31), facilitator’s experience and support is a key factor in the delivery of IPE. Another difference in the implementation was that the workshop was voluntary for speech-language pathology students, but obligatory for students of nutrition therapy and counseling and physiotherapy students, possibly resulting in greater engagement in learning activities and larger gains for the voluntarily participating speech-language pathology students (8, 31). Nevertheless, physiotherapy students also benefited significantly on both the UWE-IP-D and the in-house questionnaire.

### 4.4. Strength and limitations, future research

The current pilot study expanded IPE in the under-researched area of dementia care and included the previously neglected therapy professions of speech-language pathology, nutrition therapy and counseling, and physiotherapy, which is necessary to account for more professional diversity (6), and in the German context of academization efforts in non-medical education programs (5, 26, 82). However, the inclusion of other key dementia care professions would be desirable. Since the curricular implementation of IPE in dementia care and in general is usually a time-consuming process with many challenges and adjustments in program development and evaluation (6, 45, 51), projects and pilot studies are needed to fine-tune and improve the content, group compositions and logistics in educational programs. Nevertheless, the aim should be to move away from the project level toward the curricular implementation of IPE, that starts early and is evaluated over the long term (1, 6). Furthermore, since there is a lack of evidence to what extend IPE transfers into clinical practice, future research should examine changes in behavioral, organizational and patient outcomes (1, 6, 31).

To address the complexity of assessing interprofessional collaboration competencies, we used several assessment methods aligned with our educational goals and content, including the UWE-IP-D (37), to allow for comparison across studies. Self-assessments can provide insight into internal states (e.g., attitudes), but there are concerns, such as the veracity of self-reports, and their weaknesses in measuring knowledge and skills (31, 77). For further evaluation, objective assessment of knowledge and skills should be included, although some interprofessional competencies are difficult to assess in a standardized way (6). The applied learning methods (e.g., case-based learning, simulated case-conference, peer-assisted learning) were suitable for achieving the desired learning goals. In addition, the use of hybrid methods should be explored, as this offers flexible teaching and learning opportunities to extend the workshop in terms of content and time to gradually develop collaborative competencies (6, 45).

There are some methodological limitations to consider: we had a relatively high number of missing data, so we cannot exclude a non-respondent bias. Our sample was small for comparison between professional groups, thus challenging findings of significance. Moreover, our group was heterogenous in terms of semester numbers, number of students and facilitators from different disciplines, and voluntary/obligatory participation, which may have influenced the results. Number of semesters was confounded with the professional affiliation. Therefore, these effects cannot be separated clearly. Because our intervention is complex, the outcome can be influenced by many variables (83, 84). Therefore, we detailed the main components of our intervention in accordance with the checklist of Meinema (83) to enable a replicable design. A control group was not implemented for organizational reasons. However, a more rigorous design would be desirable to compare a uniprofessional intervention to an interprofessional one (84). The research design could further be strengthened by including a follow-up some weeks after the workshop to assess stability of learning gains.

All these aspects should be considered in future research, to raise the level of evidence and to draw conclusions about interprofessional learning and socialization processes, optimal alignment of workshop objectives, contents, methods, and competency-based assessment formats as well as differences in outcome between the professions.

### 4.5. Conclusion

In conclusion, this pilot-study confirms the effectiveness of IPE to promote interprofessional dementia care competencies and to develop positive attitudes toward interprofessional learning and collaboration in the therapy professions, thus increasing professional diversity in IPE research. In the future, the delivery of our dementia-care workshop should be expanded to a larger group of participants belonging to different professions, include additional, objective competency-based assessment methods, and be placed in the context of a longitudinal, curriculum-based IPE framework to prepare graduates for high quality patient care.

## Data availability statement

The raw data supporting the conclusions of this article will be made available by the authors, without undue reservation.

## Author contributions

KD, IA, AL, HG, TH, TT, and MG contributed to the conception and design of the study, evaluated the data, and wrote the manuscript. MS supported the statistical analysis. CH prepared the data and organized the database. All authors contributed to the article and approved the submitted version.

## Funding

This research was funded by the MG Rhineland-Palatinate (Germany), grant NeurodegX. Furthermore, funding was provided by the European Commission under the framework programme of the European Union (grant agreement No. 211696) LipiDiDiet; the EU Joint ProgrammeNeurodegenerative Disease Research (JPND) and BMBF grants Multi-MeMo (01ED2306) and EURO-FINGERS (01ED2003), and the BMBF grants Health.AI 03WIR5602B und 03WI5604B.

## Conflict of interest

The authors declare that the research was conducted in the absence of any commercial or financial relationships that could be construed as a potential conflict of interest.

## Publisher’s note

All claims expressed in this article are solely those of the authors and do not necessarily represent those of their affiliated organizations, or those of the publisher, the editors and the reviewers. Any product that may be evaluated in this article, or claim that may be made by its manufacturer, is not guaranteed or endorsed by the publisher.
